# PRELIMINARY EFFICACY OF A MULTICOMPONENT INTERVENTION DESIGNED FOR LONG-DISTANCE FAMILY CAREGIVERS

**DOI:** 10.1093/geroni/igae098.2362

**Published:** 2024-12-31

**Authors:** Verena Cimarolli, Richard Chunga, Kathrin Boerner, Sara Czaja

**Affiliations:** LeadingAge, Washington, District of Columbia, United States; University of Massachusetts Boston, Boston, Massachusetts, United States; Carl von Ossietzky University of Oldenburg, Oldenburg, Niedersachsen, Germany; Weill Cornell Medicine/Center on Aging and Behavioral Research, New York, New York, United States

## Abstract

Research on family caregiving to older adults with dementia has produced a number of evidence-based interventions designed to reduce caregiver burden and negative mental health consequences of caregiving. However, almost all existing research has focused on geographically close caregivers and evidence-based interventions were designed and tested with these proximate caregivers, not considering the unique circumstances of long-distance family caregivers (LDCs) who live more than two hours away from their care recipient. Long-distance caregiving is a common and growing phenomenon. Like geographically close caregivers of persons living with dementia, LDCs experience significant caregiving burden and strains. Yet, there are no interventions designed to address the specific needs of LDCs and to reduce caregiver burden among LDCs. This project developed and feasibility tested such an intervention called LDCare - a 7-session remotely delivered multi-component intervention combining psychoeducation with peer support designed to reduce burden in LDCs. Forty LDCs participated in the intervention and feasibility study which used a one-arm pre-post-intervention trial design and administered several caregiver burden and strain measures pre- and immediately post-intervention. Non-parametric equivalents of paired t-tests (Wilcoxon tests) were run to determine changes on burden/strain measures from pre- to post-intervention. Results show significant reductions from pre- to post-intervention in caregiver burden (Zarit Burden Interview-12), family conflict, job caregiving conflict, and role captivity (Pearlin Scales), and in a 9-item scale assessing difficulty of providing care. This study provides preliminary evidence for the efficacy of a multi-component intervention specifically designed for the needs of LDCs and for reducing burden among LDCs.

